# Effect of Muller's muscle-conjunctival resection on the upper eyelid crease position in Asian eyelids: a retrospective cohort study

**DOI:** 10.1186/s12886-022-02605-6

**Published:** 2022-09-21

**Authors:** Hung-Ju Chen, Chun-Yuan Wang, Yu-Fang Huang, Yu-Chieh Wu, Li-Chen Wei

**Affiliations:** Department of Ophthalmology, Taichung Veterans General Hospital, No. 1650, Sec. 4, Taiwan Blvd., Xitun Dist, Taichung, 407 Taiwan (R.O.C.)

**Keywords:** Müllerectomy, Marginal reflex distance, Crease height, Tarsal platform show, Blepharoptosis

## Abstract

**Purpose:**

Investigating the effect of Muller’s muscle-conjunctival resection (MMCR) on the eyelid crease position.

**Methods:**

This retrospective study included patients with unilateral acquired blepharoptosis who underwent MMCR during October 2018–December 2021. The following factors were recorded: preoperative, after phenylephrine, postoperative marginal reflex distance1 (MRD1) and tarsal platform show (TPS) of bilateral eyelids. The primary outcome was to measure the change in TPS and evaluate the factors associated with post-operative TPS. The secondary outcomes included exploring the rate of MRD1 and TPS symmetry after the operation.

**Results:**

Forty patients were included in the final analysis. The mean MRD1 of the ptotic eye was 1.28 ± 0.78 mm, 2.79 ± 0.66 mm and 3.20 ± 0.67 mm before, after phenylephrine and after the operation, respectively. The mean TPS of the ptotic eye was 5.90 ± 1.86 mm, 3.96 ± 1.49 mm and 2.79 ± 1.63 mm before, after phenylephrine and after the operation, respectively. Changes in mean TPS after the phenylephrine test and post-operation were statistically significant (*p* < 0.001). The linear regression model revealed that the absolute change in TPS after phenylephrine drop and absolute change in MRD1 post-operation were significantly correlated with the absolute change in TPS post-operation. Besides, the ratio of symmetry in MRD1 and TPS was greatly improved post-operation (82.5% and 70.0% respectively).

**Conclusion:**

MMCR is an effective surgical method for ptosis correction as it can not only correct the eyelid crease position but also narrow the wide TPS. This method is particularly beneficial to patients with both mild to moderate ptosis and an asymmetric crease height.

**Supplementary Information:**

The online version contains supplementary material available at 10.1186/s12886-022-02605-6.

## Introduction

Acquired blepharoptosis is one of the most commonly encountered upper eyelid disorders in clinical practice. Its prevalence rate ranges is 11.5% in the British adults [1], 4.7% in Iranian adults [2] and 13.5% in Korean adults [3]. Among all types of acquired blepharoptosis, involutional changes with levator aponeurosis dehiscence from the anterior tarsal plate or stretching of the aponeurosis account for the most common causes [4].

Patients with unilateral or bilateral droopy upper eyelids may complain of a tired appearance, deficits in their superior visual field and difficulty reading due to a narrow palpebral fissure on downgaze. The negative impact on an individual's quality of life depends on the degree of pupil blockage. The standard clinical management for ptosis is surgical intervention with numerous surgical options that are currently available. The Muller’s muscle-conjunctival resection (MMCR), which was first introduced by Putterman and Urist in 1975, has been proven to be an effective surgical technique for correcting a varying degree of ptosis in patients with good levator muscle function who have a great preoperative response to phenylephrine drop testing [5].

Various algorithms for different treatment goals in MMCR have been previously described [5–7]. From a functional perspective, the goal of MMCR is to increase the margin to reflex distance1 (MRD1) [8], and thus improve the superior visual field. From a cosmetic perspective, the goals of MMCR are to achieve a smooth, normal contour of the eyelid, symmetry of the marginal MRD1 and symmetry of the tarsal platform show (TPS). To date, numerous studies have explored changes in MRD1, by especially focusing on the symmetry of MRD1 after surgery, whereas less attention has been devoted to changes in TPS after the surgery [9, 10]. In fact, Goldberg et al*.* stated that the symmetry of TPS may be more important in the perception of facial appearance compared to the symmetry of MRD1 [11]. However, compared with the external levator approach in which the surgeon is able to adjust the crease of the lid intraoperatively, it is difficult to predict changes in eyelid crease during the MMCR procedure.

To address this issue, the objective of the present study was to investigate the factors associated with the post-operative TPS position, and to assess whether changes in the eyelid crease could be predicted before the operation. Furthermore, we also investigated the symmetry of MRD1 and TPS after MMCR, which are also important factors of surgical success.

## Methods

This is a retrospective, single-centre study that was approved by the Institutional Review Board of the Taichung Veterans General Hospital (CE22058A). The need for informed consent from study participants was waived by Institutional Review Board of the Taichung Veterans General Hospital and the study procedures adhered to the tenets of the Declaration of Helsinki. Patients with blepharoptosis received MMCR by a single oculoplastic surgeon (L.C.W.) at the Taichung Veterans General Hospital, and they were consecutively enrolled from October 2018–December 2021. Data were collected from electronic patient files. Parameters retrieved were patients’ characteristics (age and gender), levator function, preoperative MRD1, preoperative TPS, post-phenylephrine MRD1, post-phenylephrine TPS, MRD1 at postoperative 1 month, and TPS at postoperative 1 month.

Inclusion criteria comprised the following: 1) age > 18 years, 2) unilateral ptosis with good levator muscle function (≥ 10 mm), 3) positive response to 10% phenylephrine drop testing, 4) follow-up at least longer than 3 months. All patients included in our study were categorised into acquired blepharoptosis, and were of the aponeurotic type. Exclusion criteria comprised the following: 1) any previous eyelid trauma or surgery, 2) with contralateral eye surgery within postoperative 1 month, 3) history of systemic disease that could potentially affect eyelid position. Patients with insufficiently recorded data were also excluded from our study.

### Clinical assessment

MRD1 and TPS measurements were performed before instillation of phenylephrine drops, 10 min after instillation of phenylephrine drops, and at 1 month after the operation. MRD1 was defined as the measurement from the light reflex on the patient’s cornea to the level of the centre of the upper eyelid margin in millimetres. TPS was defined as the measurement from the centre of the upper eyelid margin to the skin fold in the vertical direction in millimetres. If no skin fold was present, it was measured to the eyelid crease. All measurements were performed by a single experienced doctor (L.C.W.) and made bilaterally to the nearest 0.5 mm using a caliper. The eyes were in the primary position of gaze for each measurement, and the patients and the observer (L.C.W.) sitting at the same level. To measure the MRD1 and TPS, the patients were asked to look straight ahead and fixed on the letters on the wall-mounted Snellen eye chart. A positive phenylephrine test result was defined as an increase in MRD1 ≥ 0.5 mm after instillation of phenylephrine drops [12]. Lid height symmetry was graded as good symmetry (≤ 0.5 mm MRD1 difference), fair symmetry (0.5 mm < MRD1 difference ≤ 1 mm) and asymmetry (MRD1 difference > 1 mm). Lid crease symmetry was graded as symmetry (≤ 1 mm TPS difference) and asymmetry (TPS difference > 1 mm). All cases received 8 mm, 12 mm MMCR or 12 mm MMCR + 1 mm tarsectomy depending on the degree of response to phenylephrine drops testing. Our target MRD1 was 2 mm below the superior limbus. Specifically, an 8-mm resection was performed in patients with the desired degree (≤ 0.5 mm under-correction) of lid height in primary position of gaze, whereas a 12-mm resection was performed in patients with 0.5 mm < lid height ≤ 1.5 mm under-correction after instillation of phenylephrine drops in primary position of gaze. Furthermore, a 12-mm MMCR + 1 mm tarsectomy was performed in patients with > 1.5 mm lid height under-correction after instillation of phenylephrine drops in primary position of gaze.

### Surgical technique

The patients were operated under topical and local anaesthesia. The upper eyelid palpebral conjunctiva and the skin of the upper eyelid were first injected with 2% lidocaine with 1:100,000 Epinephrine. The upper eyelid was everted over a Desmarres retractor, and three stitches of traction sutures with white silk thread were made centrally, medially and laterally over the upper palpebral conjunctiva. Later, a marking pen was used to mark on the conjunctiva at 4 or 6 mm away from the traction suture site. Additional marking for tarsectomy may be used on the tarsus at 1 mm away from the superior tarsal border. Three traction sutures were then pulled simultaneously ventrally towards the ceiling, and a Putterman ptosis clamp (Bausch and Lomb Storz Instruments, Manchester, MO, USA) was applied to entrap the tissues of conjunctiva and Muller’s muscle. A 6–0 polypropylene suture was passed in a running horizontal mattress pattern to the lateral end 1 mm under the clamp. The clamped tissues were cut free along the clamp border with Wescott scissors. The suture was then passed back through the palpebral conjunctiva, and exiting through the skin near the medial and lateral edge. Finally, the suture knot was fixed externalised onto the skin. All the patients were instructed to came back to outpatient clinic to remove the 6.0 polypropylene suture at day 5 postoperatively.

### Statistical analysis

The data obtained were analysed using SPSS 25 (SPSS Inc, Chicago, IL, USA). We used the Shapiro–Wilk test to determine whether the variable had a normal distribution or not. Descriptive statistics were used to study the patient’s characterisation. Categorical data were presented as percentage and continuous data as mean, standard deviation (SD) and median, interquartile range. Mann–Whitney U test was used to compare the levator function, MRD and TPS of ptotic and fellow eyes. Wilcoxon signed-rank test was used to compare statistically significant differences in MRD1 and TPS before, after phenylephrine test and after operation. Multivariate linear regression was performed to evaluate the factors that could predict and were associated with post-operative TPS after checking the normal distribution of change in TPS after the operation. In addition, Pearson correlation analysis was used to assess the relationship between the absolute change in TPS after the operation and the absolute change in MRD1 after the operation, but also the relationship between the absolute change in TPS after the operation and the absolute change in TPS after the phenylephrine test. A *p-value* of < 0.05 was accepted as the threshold of statistical significance.

## Results

### Demographics and results of initial evaluation

**Fig. 1:**
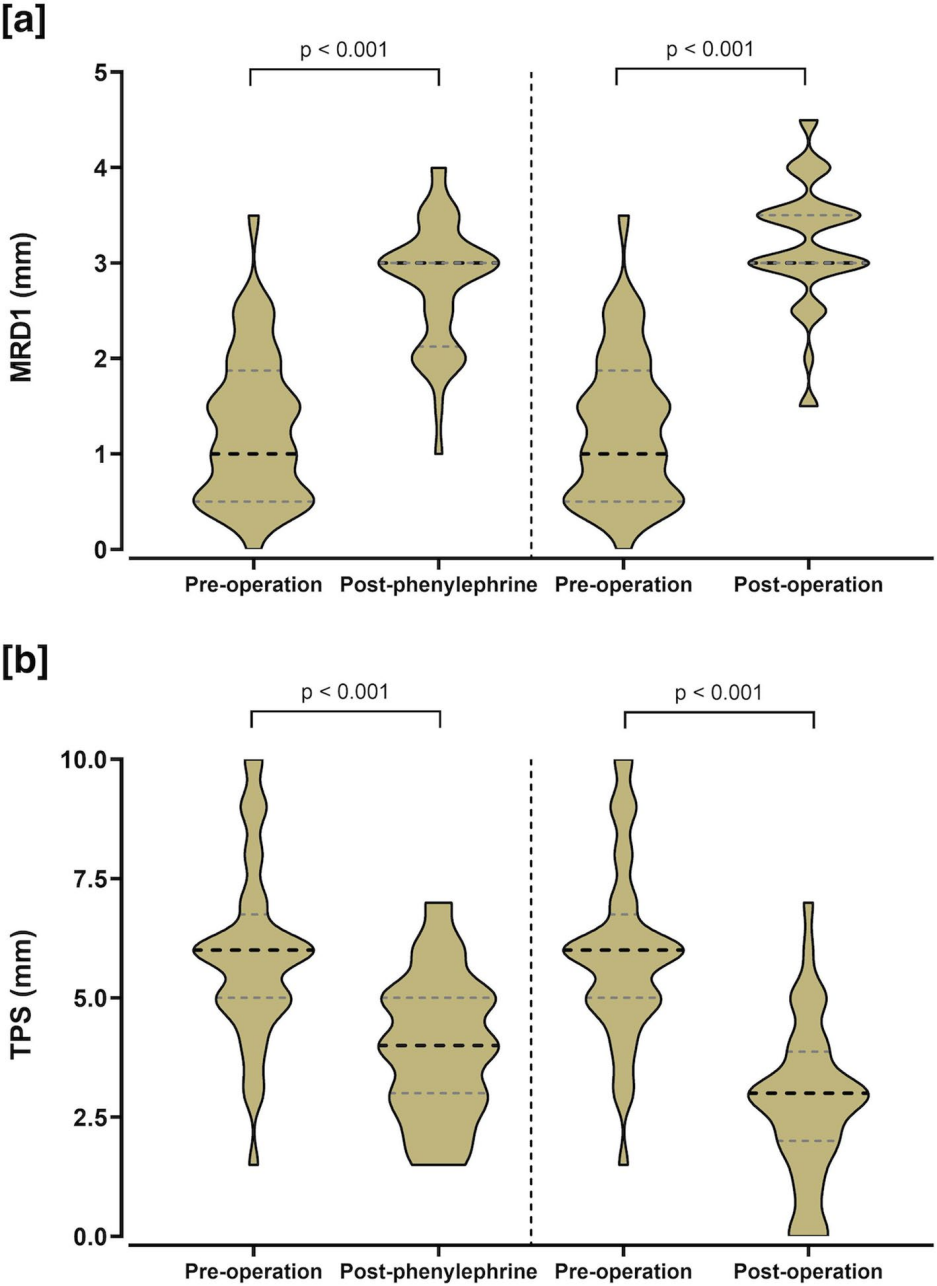
**a** Mean MRD1 of ptotic eye before operation, after phenylephrine and after operation. **b** Mean TPS of ptotic eye before operation, after phenylephrine and after operation. MRD1 = marginal reflex distance1; TPS = tarsal platform show

A total of 40 eyes from 40 patients (83.8% female, 16.2% male) aged 44.89 ± 15.42 years on average were included in this analysis. In our study, all cases were diagnosed with acquired unilateral upper eyelid ptosis and received unilateral surgery. The amount of resection depended on the severity of eyelid drooping (blepharoptosis). Thirty patients received 8-mm MMCR, seven patients received 12-mm MMCR and three patients received 12-mm MMCR + 1-mm tarsectomy. As shown in Table 1 and Fig. 1, the average levator function of the ptotic and the normal fellow eyes were 11.94 ± 1.35 mm and 13.00 ± 1.41 mm, respectively (*p* = 0.040). The mean MRD1 of the ptotic eye improved from 1.28 ± 0.78 mm to 2.79 ± 0.66 mm after the phenylephrine test (*p* < 0.001), and from 1.28 ± 0.78 mm to 3.20 ± 0.67 mm after the operation (*p* < 0.001). The mean TPS of the ptotic eye decreased from 5.90 ± 1.86 mm to 3.96 ± 1.49 mm after the phenylephrine test (*p* < 0.001), and from 5.90 ± 1.86 mm to 2.79 ± 1.63 mm after the operation (*p* < 0.001). Besides, the mean MRD1 of the normal fellow eyes decreased from 3.18 ± 0.74 mm to 2.89 ± 0.84 after the operation (*p* = 0.008), and the mean TPS of the normal fellow eyes increased from 3.31 ± 1.53 mm to 3.40 ± 1.55 mm after the operation (*p* = 0.109).

**Table 1:** Mean values and standard deviation of margin-reflex distance-1, tarsal platform show, levator function, before, after a phenylephine drop and after operation in both the ptotic eye and the fellow eye

	Ptotic eye (*n* = 40)		Normal eye (*n* = 40)		*p*-value
	Mean ± SD	median(IOR)	Mean ± SD	median(IOR)	
Levator function (mm)	11.94 ± 1.35	12.0 (10.5–13.0)	13.00 ± 1.41	13.0 (12.0–14.0)	0.040
Pre-operation MRD1 (mm)	1.28 ± 0.78	1.0 (0.5–1.9)	3.18 ± 0.74	3.0 (3.0–3.5)	< 0.001
Post-phenylephrine MRD1 (mm)	2.79 ± 0.66	3.0 (2.1–3.0)	3.10 ± 0.86	3.0 (3.0–3.5)	0.036
Post-operation MRD1 (mm)	3.20 ± 0.67	3.0 (3.0–3.5)	2.89 ± 0.84	3.0 (2.6–3.5)	0.135
Pre-operation TPS (mm)	5.90 ± 1.86	6.0 (5.0–6.8)	3.31 ± 1.53	3.0 (2.0–4.8)	< 0.001
Post-phenylephrine TPS (mm)	3.96 ± 1.49	4.0 (3.0–5.0)	3.31 ± 1.53	3.0 (2.0–4.8)	0.064
Post-operation TPS (mm)	2.79 ± 1.63	3.0 (2.0–3.9)	3.40 ± 1.55	3.0 (2.0–5.0)	0.141

*MRD1* Marginal reflex distance 1, *TPS* Tarsal platform show, *IQR* Interquartile range

### Factors associated with post-operative TPS

As summarised in Table 2, the multivariate linear regression analysis showed that the absolute change in TPS after the operation was significantly associated with the absolute change in TPS after phenylephrine drops testing (*p* < 0.001) and the absolute change in MRD1 after the operation (*p* = 0.011). However, the absolute change in TPS after the operation was not correlated with sex (*p* = 0.658), age (*p* = 0.403), resection length (*p* = 0.637), and the absolute change in MRD1 after phenylephrine (*p* = 0.881).

**Table 2:** Multivariate linear regression analyses among various clinical factors for the change of TPS after operation

	Unstandardized coefficients		Standardized coefficients	95% CI for B		
Predictor Valuables	B	SE	β	Lower bound	Upper bound	*p*-value
Age	-0.011	0.014	-0.107	-0.039	0.016	0.403
Sex	0.237	0.532	0.056	-0.846	1.320	0.658
Resection length	-0.050	0.105	-0.018	-0.263	0.163	0.637
Change of TPS after phenylephrine (mm)	-0.997	0.158	-0.670	-1.318	-0.676	< 0.001
Change of MRD1 after phenylephrine (mm)	0.060	0.396	-0.126	-0.746	0.865	0.881
Change of MRD1 after operation (mm)	0.784	0.290	0.368	0.195	1.374	0.011

*MRD1* Marginal reflex distance 1, *TPS* Tarsal platform show, *SE* Standard error, *CI* Confidence interval

**Fig. 2:**
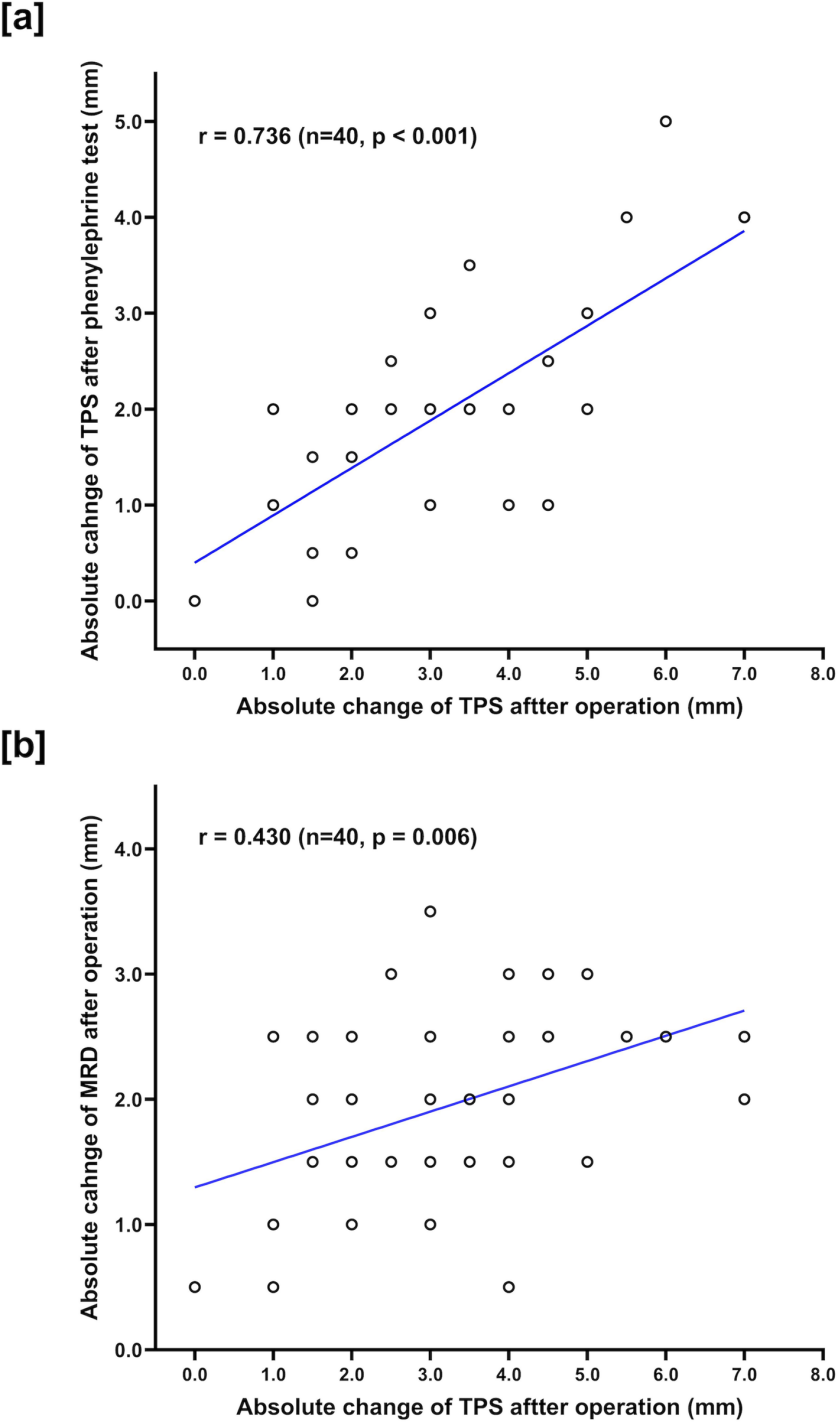
**a** Pearson correlation tests between the absolute change of TPS after operation and the absolute change of TPS after phenylephrine test. **b** Pearson correlation tests between the absolute change of TPS after operation and the absolute change of MRD1 after operation. TPS = tarsal platform show; MRD1 = marginal reflex distance1

Additionally, the post-operative correlation analysis showed in Fig. 2 confirmed the strong correlation between the absolute change in TPS after the operation and the absolute change in TPS after phenylephrine test (*r* = 0.736, *p* < 0.001) and a moderate correlation between the absolute change in TPS after the operation and the absolute change in MRD1 after the operation (*r* = 0.430, *p* = 0.026).

### Lid height and lid crease symmetry after phenylephrine drops testing or post-operation

**Fig. 3:**
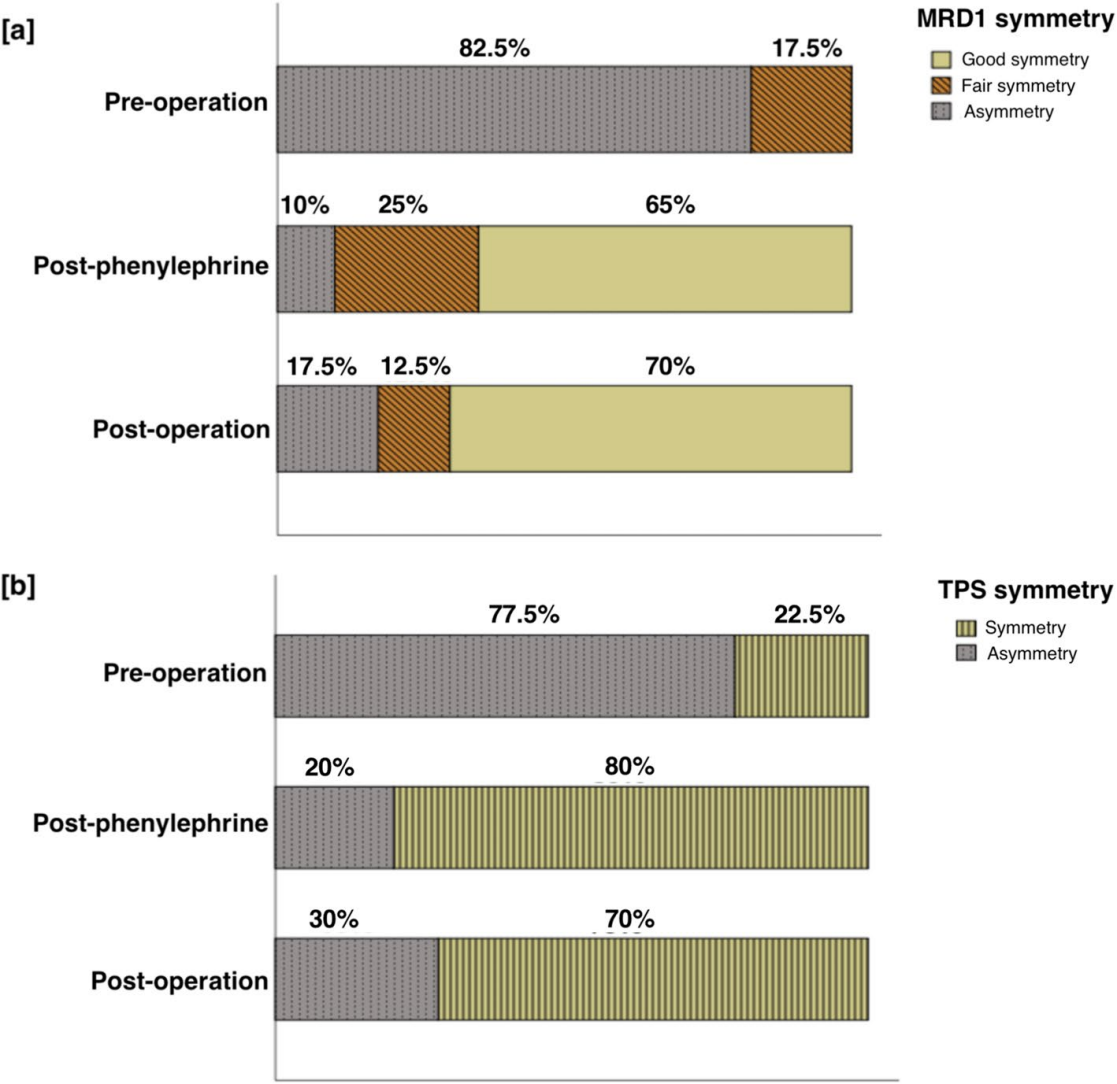
**a** The symmetry rate of MRD1 before, after phenylephrine drop, after operation. **b** The symmetry rate of TPS before, after phenylephrine drop, after operation. MRD1 = marginal reflex distance1; TPS = tarsal platform show

**Fig. 4:**
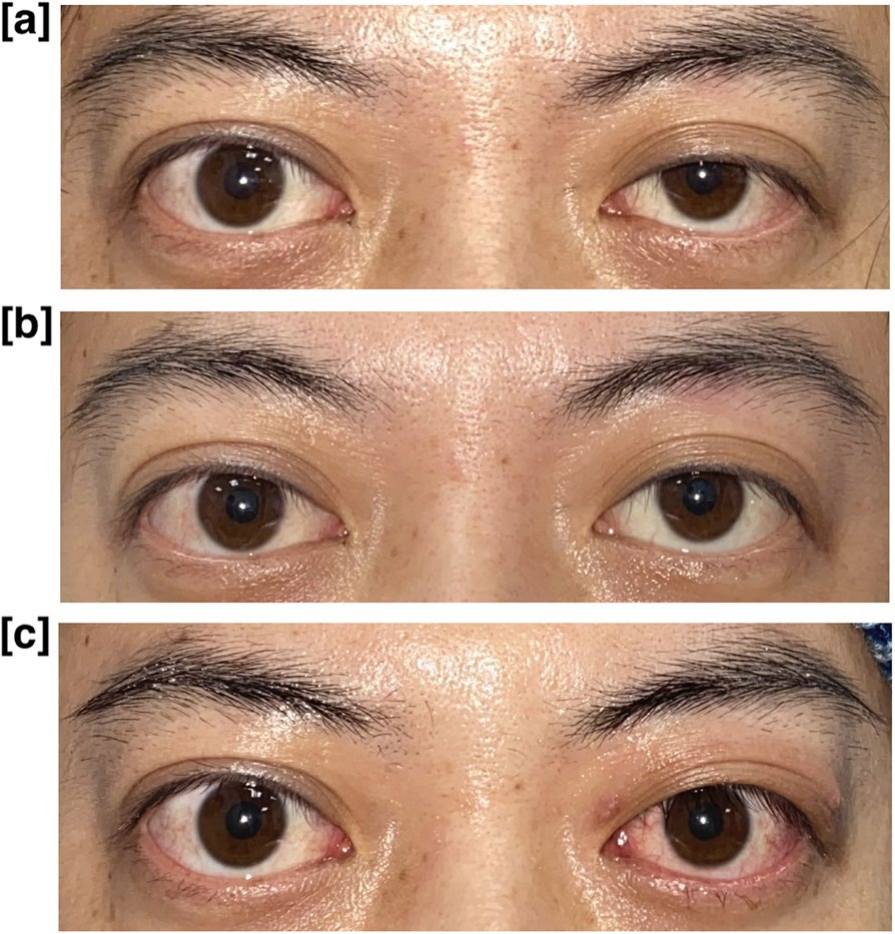
**a** Preoperative MRD1 and TPS difference (**b**) MRD1 and TPS difference after phenylephrine test (**c**) MRD1 and TPS difference after MMCR. MRD1 = marginal reflex distance1; TPS = tarsal platform show

Figure 3 shows the proportion of patients with lid height and lid crease symmetry or asymmetry. The proportion of patients with symmetrical lid height (MRD-1 difference ≤ 1 mm) increased from 17.5% to 90.0% after phenylephrine drop testing, and from 17.5% to 82.5% after the operation. Similarly, the proportion of patients with symmetrical lid crease (TPS difference ≤ 1 mm) increased from 22.5% to 80% and from 22.5% to 70% after phenylephrine testing and after the operation, respectively. Of note, three of 40 patients (7.5%) had symmetric TPS before the operation but asymmetric TPS after the operation. Five of 40 patients (12.5%) in our study developed apparently contralateral ptosis after the operation and needed further operation for contralateral eye. No post-operative complications, such as entropion, ectropion, lagophthalmos or exposure keratopathy were reported. Representative pre-operative, post-phenylephrine test and post-operative images are shown in Fig. 4.

## Discussion

This study sought to further explore the TPS change in ptotic patients undergoing 10% phenylephrine test and MMCR. A number of previous studies have improved the authors’ understanding of the phenylephrine test and MMCR processes since they were first proposed by Putterman and Urist [5, 7, 9]. These studies focused on using phenylephrine drop test results in order to refine their approach of ptosis repair by primarily assessing the effect of MRD1 change. However, less attention has been devoted to the effect of the TPS position and TPS symmetry, which are both pivotal from an aesthetic perspective. In our study, we found that TPS was dramatically decreased after instillation of phenylephrine drops and after the operation. A linear regression model revealed that the absolute change in TPS after instillation of phenylephrine drops and the absolute change in MRD1 after the operation had a significant effect on the absolute change in TPS after the operation (*p* < 0.001 and *p* = 0.011 respectively). Besides, the ratio of patients with symmetric TPS was also greatly improved after instillation of phenylephrine drops and after the operation (82.5% and 70.0% respectively).

TPS is a key measurement for quantifying aesthetically the findings of the upper face. TPS is determined by the vertical distance between the centre upper eyelid margin and the skin fold in primary position of gaze. A weak posterior lamella from a ptotic eyelid, including the Muller’s muscle and the levator aponeurotic tarsal insertion, would result in a greater strain in the anterior muscle–skin aponeurotic insertion, which manifests clinically as a higher TPS [13]. In our study, we found that TPS was dramatically decreased in ptotic eyes after instillation of phenylephrine drops and MMCR. The exact mechanism of lower TPS remains to be elucidated. Lew et al*.* [14] stated that the shortening of TPS after instillation of phenylephrine drop and MMCR could be mainly explained by two factors. First, the eyelid itself decreases after vertical shortening of the posterior lamella so that the tarsal platform is tucked up under the orbital fat and the eyelid skin. Second, elevation of the eyelid margin induces relaxation of eyebrow compensatory drive, which allows the eyebrow to descend in a way that the eyebrow fat pad fills the superior sulcus and hides part of the tarsal platform. Lee et al. stated that vertical shortening of the posterior lamella, plication or advancement of the Muller’s muscle after MMCR may also include some degree of plication of the posterior aspect of the levator aponeurosis that consequently results in TPS shortening [15]. Additionally, Marcet et al.[16] reported that advancement of the recessed levator aponeurosis to the superior tarsal border is another reason for the changes in TPS after MMCR.

In addition to understanding the mechanism that facilitates changes in TPS, the accurate prediction of the post-operative TPS before the operation has also been an important issue to the surgical design. However, accurate prediction is difficult. Multiple factors come into play, including eyelid and eyebrow compensatory mechanisms [17], volume of the eyebrow fat pad [18], volume of orbital fat, skin elasticity and thickness [19], variation in tarsus morphology [20] and bony symmetry [21, 22]. For optimal results, surgical decision making processes should incorporate these multiple factors despite the fact that some of them can be difficult to predict. In our study, we found that the absolute change in TPS after instillation of phenylephrine drops could be a well-predictable factor of post-operative TPS, whereas the resection length in MMCR could not accurately predict post-operative TPS. The possible reason to explain this is that in phenylephrine-response blepharoptosis patients, phenylephrine drop stimulates the contraction of the sympathetic innervation of the Muller’s muscle, which mimics the surgical effect of shortening the Muller’s muscle, and which subsequently results in eyelid elevation and TPS reduction. Furthermore, patients undergoing longer tissue resection had a less desirable response to phenylephrine drops initially, thereby longer tissue resection was necessary to achieve the desired outcome.

As for the symmetry analysis of MRD1 and TPS, we found that they were both less symmetrical after the operation compared to after the phenylephrine test. There are several reasons that could explain this finding. First, our treatment goals focused on targeting MRD1 rather than symmetric TPS. The amount of resection depended on the severity of eyelid drooping rather than the symmetry of the eyelid, which may have resulted in more asymmetric results. Second, the Hering’s dependence test may not have truly reflected normal eyes during the phenylephrine test. Both anecdotal experience and a recent study have shown that despite the fact that the Hering’s law was negative after the phenylephrine test, some patients could still experience appearance asymmetry and a decrease in MRD1 and postoperatively [23]. Cetinkaya et al*.* explained that the Hering’s effect might be suppressed by the frontalis muscle action of the fellow eye during pre-operative evaluation [23]. However, due to its retrospective nature, it is not possible to comment on the precision with which this test was conducted in patients that could strongly suppress the Hering’s effect. Third, the eyelid level may be affected depending on which eye is the dominant one. Lyon et al.[24] instilled 2.5% phenylephrine in 54 eyes with unilateral or asymmetric ptosis and found that the contralateral lid droop was significantly more common when ptosis was greater in the dominant eye.

Besides, among the patients with post-operative asymmetric TPS in our study, three of whom had symmetric TPS before the operation whereas came out with asymmetric TPS after the operation. Basically, TPS should be initially longer on the more ptotic side. The reason for initially symmetric TPS in unilateral ptosis may be bony asymmetry [21, 22], soft tissue asymmetry[18, 19], eyebrow asymmetry [25], or strong eye dominance [26] on the contralateral side. With a view to achieving a more successful cosmetic outcome, Goldberg et al.[11] stated that ptosis surgery might be better combined with asymmetric blepharoplasty surgery in patients with initially symmetric TPS in order to increase the TPS on the more crowded side. Regarding patients with longer TPS in their ptosis eye, they may only need ptosis surgery to improve TPS symmetry.

Furthermore, another important finding in our study was that some patients developed drooping of the fellow upper eyelid, and even five of the 36 patients (13.9%) developed measurable drooping, ≥ 1 mm MRD-1 reduction, after successful unilateral MMCR. A similar result was found by Erb et al.[27] in a study where the authors found that roughly 17% of their patients demonstrated a decrease in contralateral eyelid height of more than 1 mm, with 5% of patients requiring surgical repair during the first post-operative year after ptosis surgery. This phenomenon can be explained by Hering’s law in that equal and simultaneous innervations are sent to paired yoke muscles [28, 29]. The postoperative decrease in the upper eyelid height of the contralateral eye was a manifestation of a previously masked, subclinical blepharoptosis, which was attributable to Hering law. Hence, the identification of patients with occult bilateral ptosis is important in the pre-operative evaluation to avoid unexpected outcomes. In fact, several preoperative tests, such as occlusion of the ptotic eye, manual elevation of the ptotic eye and the phenylephrine test, have been widely used [30]. Among these methods, the manual elevation test is reported to be the most effective, whereas the phenylephrine test is more sensitive to mild ptosis [31].

There are several limitations to this study. First, the retrospective nature of our data analysis and the limited number of patients included in this study. Second, patients included in our study were all of Asian heritage and ethnicity. As we know, the anatomy of the eyelid and periorbital structures are different among different ethnicities. For example, the Asian eyelid skin is often thicker, with pretarsal and sub-orbicularis oculi muscle adipose layers that are rarely present in the Caucasian eyelid [32]. Further studies should be performed to observe if the TPS reduction and symmetric TPS improvement can also occur in patients of different ethnicity and origins, such as Caucasians, Hispanics, etc. Third, we only evaluated short-term outcomes (3-month follow-up after MMCR), and thus the long-term surgical effect remains unknown. Last, we only recorded the crease height in the primary position of gaze; however, dermachalasis, subcutaneous or eyebrow fat may mask the true change after operation. Further studies should focus on measuring the crease height both in primary and down gazes, thus allowing to evaluate the superior eyelid crease change after the phenylephrine test and ptosis surgery more clearly.

In conclusion, this study provides a simplified preoperative evaluation that allows a valid prediction of the surgical effect before the operation. From a functional perspective, the eyelid position showed notable improvement both after the phenylephrine test and the ptosis surgery. From a cosmetic perspective, the superior eyelid crease height at primary gaze was significantly decreased to a symmetrical level with the contralateral eyelid in approximately 70% of patients after phenylephrine test and MMCR. Of note, the change in eyelid crease height at primary gaze after phenylephrine was independent of the factors that were significantly correlated with the change in eyelid crease height at primary position of gaze after MMCR. Finally, MMCR is an effective and predictable surgical method. This method is particularly beneficial to patients with both mild to moderate ptosis and an asymmetric crease height.

## Supplementary Information

**Additional file 1.** This is the raw data used in our study to analyze the surgical outcomes of patient undergoing Muller’s muscle-conjunctival resection (MMCR), including resection length in surgical eyes, preoperative, post-phenylephrine, postoperative marginal reflex distance 1 (MRD1) and tarsal platform show (TPS) of bilateral eyelids.

## Data Availability

The datasets used during the current study are available in the supplementary material file [see Additional file 1].

## Abbreviations

MMCR: Muller’s muscle-conjunctival resection
MRD1: Marginal reflex distance1
TPS: Tarsal platform show

## References

[1] Sridharan GV, Tallis RC, Leatherbarrow B, Forman WM (1995). A community survey of ptosis of the eyelid and pupil size of elderly people. Age Ageing.

[2] Hashemi H, Khabazkhoob M, Emamian MH, Yekta A, Jafari A, Nabovati P, Fotouhi A (2016). The prevalence of ptosis in an Iranian adult population. J Curr Ophthalmol.

[3] Kim MH, Cho J, Zhao D, Woo KI, Kim YD, Kim S, Yang SW (2017). Prevalence and associated factors of blepharoptosis in Korean adult population: The Korea national health and nutrition examination survey 2008–2011. Eye (Lond).

[4] Lee YG, Son BJ, Lee KH, Lee SY, Kim CY (2018). Clinical and demographic characteristics of blepharoptosis in Korea: A 24-year experience including 2,328 patients. Korean J Ophthalmol.

[5] Putterman AM, Urist MJ (1975). Müller muscle-conjunctiva resection: technique for treatment of blepharoptosis. Arch Ophthalmol.

[6] Dresner SC (1991). Further modifications of the Müller's muscle-conjunctival resection procedure for blepharoptosis. Ophthalmic Plast Reconstr Surg.

[7] Perry JD, Kadakia A, Foster JA (2002). A new algorithm for ptosis repair using conjunctival Müllerectomy with or without tarsectomy. Ophthalmic Plast Reconstr Surg.

[8] Putterman AM (2012). Margin reflex distance (MRD) 1, 2, and 3. Ophthalmic Plast Reconstr Surg.

[9] Ben Simon GJ, Lee S, Schwarcz RM, McCann JD, Goldberg RA (2007). Muller's muscle-conjunctival resection for correction of upper eyelid ptosis: relationship between phenylephrine testing and the amount of tissue resected with final eyelid position. Arch Facial Plast Surg.

[10] Couch SM (2016). Correction of eyelid crease asymmetry and ptosis. Facial Plast Surg Clin North Am.

[11] Goldberg RA, Lew H (2011). Cosmetic outcome of posterior approach ptosis surgery (an American ophthalmological society thesis). Trans Am Ophthalmol Soc.

[12] Baldwin HC, Bhagey J, Khooshabeh R (2005). Open sky Müller muscle-conjunctival resection in phenylephrine test-negative blepharoptosis patients. Ophthalmic Plast Reconstr Surg.

[13] Queirós TSM, Won-Kim H-R, Sales-Sanz A, Sales-Sanz M (2020). Effect of topical Phenylephrine on the upper eyelid crease position. Acta Ophthalmol.

[14] Lew H, Goldberg RA (2016). Maximizing symmetry in upper blepharoplasty: the role of microptosis surgery. Plast Reconstr Surg.

[15] Lee NG, Lin L-w, Mehta S, Freitag S (2015). Response to phenylephrine testing in upper eyelids with ptosis. Digit J Ophthalmol : DJO.

[16] Marcet MM, Setabutr P, Lemke BN, Collins ME, Fleming JC, Wesley RE, Pinto JM, Putterman AM (2010). Surgical microanatomy of the müller muscle-conjunctival resection ptosis procedure. Ophthalmic Plast Reconstr Surg.

[17] Hamedani AG, Gold DR (2017). Eyelid dysfunction in neurodegenerative, neurogenetic, and neurometabolic disease. Front Neurol.

[18] Chen W (1999). Aesthetic eyelid surgery in Asians: an East-West view. Hong Kong J Ophthalmol.

[19] Farber SE, Codner MA (2020). Evaluation and management of acquired ptosis. Plast Aesthet Res.

[20] Chanlalit W (2015). Variations of the double eyelid and the upper tarsus in Asians. J Med Assoc Thai.

[21] Katsumata A, Fujishita M, Maeda M, Ariji Y, Ariji E, Langlais RP (2005). 3D-CT evaluation of facial asymmetry. Oral Surg Oral Med Oral Pathol Oral Radiol Endod.

[22] Ing E, Safarpour A, Ing T, Ing S (2006). Ocular adnexal asymmetry in models: a magazine photograph analysis. Can J Ophthalmol.

[23] Cetinkaya A, Kersten RC (2012). Surgical outcomes in patients with bilateral ptosis and Hering's dependence. Ophthalmol.

[24] Wiley HJ, Son DJ: Higgins JPT, Green S. Assessing risk of bias in included studies. Cochrane Handb Syst Rev Intervent. 2008;1:187–241.

[25] Costin BR, Wyszynski PJ, Rubinstein TJ, Choudhary MM, Chundury RV, McBride JM, Levine MR, Perry JD (2016). Frontalis muscle asymmetry and lateral landmarks. Ophthalmic Plast Reconstr Surg.

[26] Lyon DB, Gonnering RS, Dortzbach RK, Lemke BN (1993). Unilateral ptosis and eye dominance. Ophthalmic Plast Reconstr Surg.

[27] Erb MH, Kersten RC, Yip CC, Hudak D, Kulwin DR, McCulley TJ (2004). Effect of unilateral blepharoptosis repair on contralateral eyelid position. Ophthalmic Plast Reconstr Surg.

[28] Zoumalan CI, Lisman RD (2010). Evaluation and management of unilateral ptosis and avoiding contralateral ptosis. Aesthet Surg J.

[29] Chen AD, Lai YW, Lai HT, Huang SH, Lee SS, Chang KP, Lai CS (2016). The impact of Hering's law in blepharoptosis: literature review. Ann Plast Surg.

[30] Schechter RJ (1978). Ptosis with contralateral lid retraction due to excessive innervation of the levator palpebrae superiorus. Ann Ophthalmol.

[31] Meyer DR, Wobig JL (1992). Detection of contralateral eyelid retraction associated with blepharoptosis. Ophthalmol.

[32] Kiranantawat K, Suhk JH, Nguyen AH (2015). The Asian eyelid: relevant anatomy. Semin Plast Surg.

