# Supplementary material for: Effect of Muller's muscle-conjunctival resection on the upper eyelid crease position in Asian eyelids: a retrospective cohort study
Source: BMC Ophthalmol. 2022 Sep 21;22:377. doi: 10.1186/s12886-022-02605-6 (PMC9490903; doi:10.1186/s12886-022-02605-6)
Supplement: Supplementary file 1 — Additional file 1. This is the raw data used in our study to analyze the surgical outcomes of patient undergoing Muller’s muscle-conjunctival resection (MMCR), including resection length in surgical eyes, preoperative, post-phenylephrine, postoperative marginal reflex distance 1 (MRD1) and tarsal platform show (TPS) of bilateral eyelids. [file 12886_2022_2605_MOESM1_ESM.docx]

**Additional files legend**

Additional file 1: This is the raw data used in our study to analyze the surgical outcomes of patient undergoing Muller’s muscle-conjunctival resection (MMCR), including resection length in surgical eyes, preoperative, post-phenylephrine, postoperative marginal reflex distance 1 (MRD1) and tarsal platform show (TPS) of bilateral eyelids.
